# Targeting *anaplastic lymphoma kinase (ALK)* gene alterations in neuroblastoma by using alkylating pyrrole-imidazole polyamides

**DOI:** 10.1371/journal.pone.0257718

**Published:** 2021-09-30

**Authors:** Yoko Ota, Hiroyuki Yoda, Takahiro Inoue, Takayoshi Watanabe, Yoshinao Shinozaki, Atsushi Takatori, Hiroki Nagase

**Affiliations:** 1 Division of Innovative Cancer Therapeutics, Chiba Cancer Center Research Institute, Chiba, Japan; 2 Graduate School of Medical and Pharmaceutical Sciences, Chiba University, Chiba, Japan; 3 Division of Cancer Genetics, Chiba Cancer Center Research Institute, Chiba, Japan; Sechenov First Medical University, RUSSIAN FEDERATION

## Abstract

*Anaplastic lymphoma kinase* (*ALK*) aberration is related to high-risk neuroblastomas and is an important therapeutic target. As acquired resistance to ALK tyrosine kinase inhibitors is inevitable, novel anti-ALK drug development is necessary in order to overcome potential drug resistance against ATP-competitive kinase inhibitors. In this study, to overcome ALK inhibitor resistance, we examined the growth inhibition effects of newly developed *ALK*-targeting pyrrole-imidazole polyamide CCC-003, which was designed to directly bind and alkylate DNA within the F1174L-mutated *ALK* gene. CCC-003 suppressed cell proliferation in *ALK*-mutated neuroblastoma cells. The expression of total and phosphorylated ALK was downregulated by CCC-003 treatment but not by treatment with a mismatch polyamide without any binding motif within the *ALK* gene region. CCC-003 preferentially bound to the DNA sequence with the F1174L mutation and significantly suppressed tumor progression in a human neuroblastoma xenograft mouse model. Our data suggest that the specific binding of CCC-003 to mutated DNA within the *ALK* gene exerts its anti-tumor activity through a mode of action that is distinct from those of other ALK inhibitors. In summary, our current study provides evidence for the potential of pyrrole-imidazole polyamide ALK inhibitor CCC-003 for the treatment of neuroblastoma thus offering a possible solution to the problem of tyrosine kinase inhibitor resistance.

## Introduction

Neuroblastoma is a pediatric cancer and the most frequently occurring extracranial solid tumor in children. The prognosis of neuroblastoma depends on risk group and stage. Patients with high-risk or stage Ⅳ neuroblastoma have a survival rate of less than 50%, despite extensive treatment [1]. The *anaplastic lymphoma kinase* (*ALK*) gene is known to be one of the factors related to the prognosis of neuroblastoma, and mutations of this gene are related to high-risk neuroblastoma. Point mutations and gene amplifications have been described in approximately 10% of patients with neuroblastoma and 14% of individuals considered as high-risk cases [2–5]. Further, gain-of-function mutations and *ALK* overexpression are associated with both familial and sporadic neuroblastoma disease [6, 7]. The overall survival of patients with mutated *ALK* is lower, making *ALK* aberrations prognostic biomarkers of lower survival in neuroblastoma [4]. Hence, ALK has been an important therapeutic target for the development of novel drugs against aggressive neuroblastoma.

A variety of malignancies harbor translocations, amplifications, or other oncogenic mutations of *ALK* [8]. In lung cancer and lymphoma, *ALK* fusion is the most common aberration. Most patients with lung cancer relapse within a few years of tyrosine kinase inhibitor (TKI) therapy due to acquired resistance [9]. In neuroblastoma, most *ALK* mutations occur within the kinase domain with two mutation hotspots at F1174 and R1275 [5, 7, 10]. Among activating mutations, F1174L has been reported to confer resistance to ALK inhibitor crizotinib in neuroblastoma [11–13]. Therefore, a novel anti-ALK drug is necessary to combat potential drug resistance to ATP-competitive TKIs in *ALK*-driven neuroblastoma.

Pyrrole-imidazole (PI) polyamides are hairpin-shaped small compounds containing pyrroles and imidazoles that bind to coding regions within the minor groove of genomic DNA in a sequence-specific manner [14, 15]. PI polyamides have a complementary code for minor groove recognition. In particular, imidazole/pyrrole recognizes G/C pairs and pyrrole/pyrrole binds to A/T pairs. An alkylating moiety, *seco*-CBI, conjugated with the N-terminal of PI polyamide, can disrupt the expression of target genes by covalently alkylating the template strand of DNA and impeding RNA polymerase activity [16, 17].

In the present study, we developed a novel *ALK*-targeting PI polyamide, CCC-003, in order to target the F1174L mutation of *ALK*, and investigated the compound’s anti-tumor activity. CCC-003 was designed to directly bind to and alkylate DNA within the F1174L-mutated *ALK* gene in neuroblastoma cells. CCC-003 exhibited anti-proliferative effects in *ALK* mutation-positive neuroblastoma *in vitro* and *in vivo*. These results suggest that CCC-003 could be an effective anti-cancer agent for the treatment of *ALK-*mutated neuroblastoma.

## Materials and methods

### Compound synthesis

**Fig 1:**
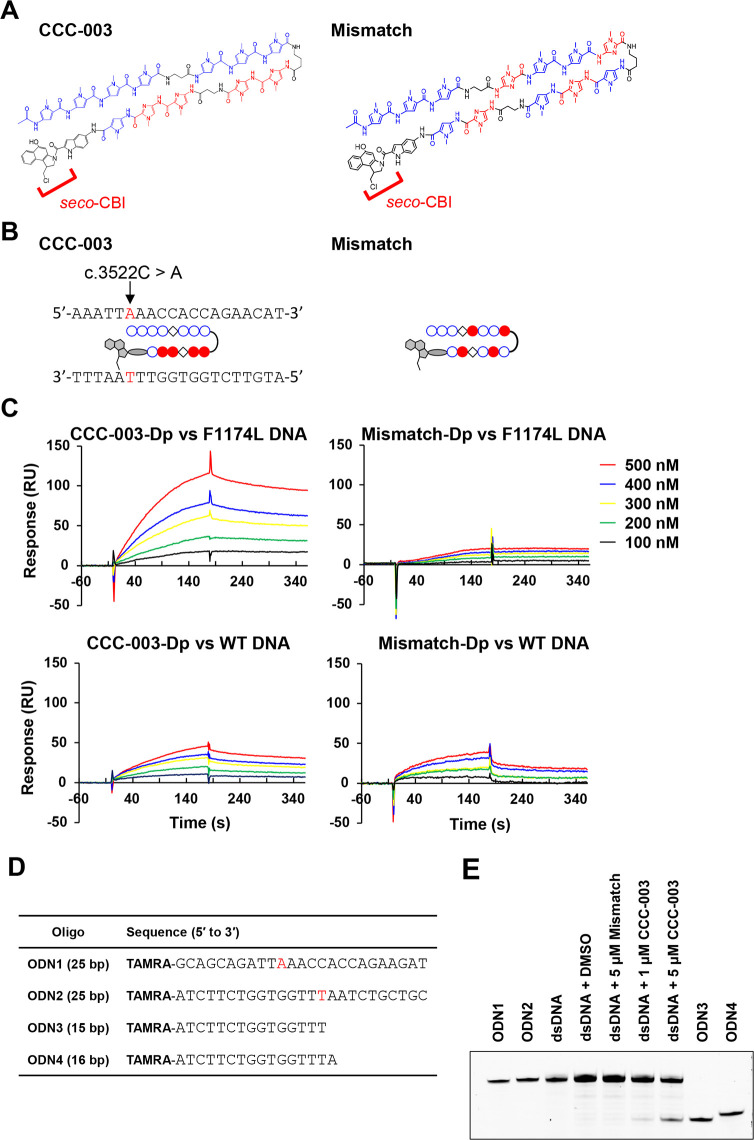
CCC-003 specifically recognizes the DNA sequence of mutant *ALK*. (A) Chemical structure of CCC-003, a sequence-specific DNA-alkylating agent directly targeting the F1174L-mutated *ALK* gene, and the mismatch polyamide (Mismatch), which had no binding motif within the coding region of the *ALK* gene. The site of alkylation is shown in red. (B) Schematic drawing of CCC-003 and Mismatch at the site of *ALK* F1174L mutation. Blue open circles represent pyrrole moieties and red circles represent imidazole. Arrow indicates the position of the *ALK* F1174L mutation. (C) SPR sensorgrams for the interaction of CCC-003-Dp with hairpin DNAs containing the F1174L mutation sequence, Mismatch-Dp with hairpin DNAs containing the F1174L mutation sequence, CCC-003-Dp with hairpin DNAs containing the wild-type sequence, and Mismatch-Dp with hairpin DNAs containing the wild-type sequence. Oligonucleotides were immobilized on the surface of an SA sensor chip. Five curves of the lowest, mid-low, middle, mid-high, and highest concentration of PI polyamide indicate 100, 200, 300, 400, and 500 nM, respectively. (D) Oligonucleotides used in PAGE analysis. (E) PAGE analysis confirmed the alkylation site on target DNA fragment by CCC-003. Annealed DNA fragment (dsDNA) using 5′-TAMRA-labeled DNA oligonucleotides (ODN1 and ODN2) was incubated with DMSO, Mismatch polyamide or CCC-003 and heated to visualize alkylated bands by PAGE analysis. The alkylated DNA fragments were analyzed with reference DNAs (ODN3 and ODN4).

All reagents and solvents were used as purchased without further purification. The manufacturers of the chemicals were as follows: Fmoc-β-alanine, Fmoc-γ-Abu-OH, and 2-Cl-Tri-Cl resin were obtained from Merck (Darmstadt, Germany). 1-Ethyl-3-(3-dimethylaminopropyl)carbodiimide hydrochloride (WSC) and O-(6-chlorobenzotriazol-1-yl)-1,1,3,3-tetramethyluronium hexafluorophosphate (HCTU) were obtained from Peptide Institute Inc (Ibaraki, Japan). HPLC-grade acetonitrile and biotin were obtained from Merck. 4-(Fmoc-amino)-1-methyl-1H-imidazole-2-carboxylic acid (Im), N,N-dimethylpropanediamine (DIEA), HCl, and N-methylpyrrolidone (NMP) were obtained from Fuji Film-Wako Chemicals (Tokyo, Japan). The solid-phase synthesis of PI-polyamides was performed using a PSSM-8 peptide synthesizer (Shimadzu, Kyoto, Japan). The LC-MS analyses were conducted on the LC-MS2020 system (Shimadzu) using a 10 mm × 150 mm Gemini-NX3u 5-ODS-H reverse-phase column (Phenomenex, Torrance, CA, USA), and the analytes were eluted by a linear gradient of 0.1% HCl in 0–100% or 30%-75% acetonitrile over 30 min with detection at 310 nm. The alkylating agent indole-*seco*-CBI was prepared following the procedures reported by Lajiness et al. [18]. PI-polyamide-indole-*seco*-CBI conjugates, CCC-003 and ALK Mismatch, were synthesized by the amide coupling of the N-acetylated polyamide backbone (Ac-NH-PyPyPyPyβPyPyPy-γ-ImImβImImPy-COOH and Ac-NH-PyPyPyβImPyPyIm-γ-PyImPyβImPy-COOH for CCC-003 and the mismatch polyamide, respectively) with indole-*seco*-CBI using WSC as the coupling reagent in NMP. Meanwhile, the synthesis of biotinylated CCC-003 was achieved in a one-pot procedure: the coupling of the N-terminal-free polyamide backbone in the presence of a large excess (10 eq) of WSC and indole-*seco*-CBI in order to obtain CCC-003. The compounds PyPyPyPyβPyPyPy-γ-ImImβImImPy-indole-*seco*-CBI (CCC-003) and PyPyPyβImPyPyIm-γ-PyImPyβImPy-indole-*seco*-CBI (Mismatch polyamide) were shown in Fig 1A. The mismatch polyamide had no binding motif within the coding region of the *ALK* gene.

### Surface plasmon resonance (SPR) assay

All SPR experiments were performed on a Biacore X100 (GE Healthcare, Marlborough, MA, USA) at 25°C as described previously [19]. A biotinylated hairpin F1174L-mutated *ALK* oligonucleotide (5’-biotin-AAATTAAACCACCAGAACATTTTTATGTTCTGGTGGTTTAATTT-3’, Hokkaido System Science, Sapporo, Japan) and an *ALK* wild-type oligonucleotide (5’-biotin-AAATTCAACCACCAGAACATTTTTATGTTCTGGTGGTTGAATTT-3’) were immobilized on a streptavidin-coated SA sensor chip at a flow rate of 10 μL/min to obtain the required level of immobilization (up to approximately 500 resonance units).

CCC-003 and mismatch polyamide were coupled with dimethylaminopropylamine (Dp) at the C-terminus (CCC-003-Dp and mismatch polyamide-Dp, respectively). Samples were dissolved in HBS-EP buffer (10 mM 4-(2-hydroxyethyl)- 1-piperazineethanesulfonic acid (HEPES), 150 mM NaCl, 3 mM ethylenediamine tetraacetic acid (EDTA), and 0.005% surfactant P20) with 0.1% DMSO at 100, 200, 300, 400, and 500 nM. HBS-EP buffer with 0.1% DMSO at 25°C was used for this study. Biacore X100 Evaluation Software ver 2.0 was used to calculate the association rate constant (*ka*), dissociation rate constant (*kd*), and dissociation equilibrium constant (KD). The binding model with mass transfer was used to fit all of the sensorgrams in order to obtain a 1:1 binding model.

### High-resolution denaturing polyacrylamide gel electrophoresis (PAGE)

The detailed method of PAGE analysis was described previously [20]. All DNA fragments were purchased from Hokkaido System Science. DNA samples were dissolved in 1 × TE buffer (pH 7.2). CCC-003 was dissolved in DMSO. ODN 1 and 2 (25 bp) were annealed in a final volume of 20 μL containing 1 μM of each strand (Fig 1D). Annealing was performed by heating to 95°C and cooling to the starting temperature of 25°C at a cooling rate of 2°C/ 2 min. For DNA alkylation, 2 μL of 1 μM or 5 μM CCC-003, 5 μM Mismatch, or DMSO was added to 4 μL of double stranded DNA fragments in a final volume of 20 μL containing 5 mM sodium phosphate buffer (pH 7.0) and incubated at 25°C for 18 h. After heating at 95°C for 25 min, 2 μL of 1 M piperidine was added to the alkylated DNA samples followed by heating at 95°C for 25 min again. The samples were recovered by vacuum centrifugation at 45°C for 15 min. The pellets were dissolved in 40 μL of denaturing buffer (formamide: 10 × loading buffer: 10 × TBE buffer: nuclease-free water = 5:1:1:3) and heated at 95°C for 25 min. The 5 μL of samples were loaded on 20% polyacrylamide gel containing 7 M of urea and electrophoresed (room temperature, 200 V, 90 min) in 1× TBE buffer. The gel was imaged on a ChemiDoc MP Imaging System (Bio-Rad, Hercules, CA, USA).

### Cell lines and cell culture

Human neuroblastoma SH-SY5Y, Kelly, and SK-N-AS cells were maintained in DMEM, RPMI1640, and MEM/F12, respectively. All cell lines were supplemented with 10% FBS (Thermo Fisher Scientific, Waltham, MA, USA) as well as penicillin/streptomycin (Thermo Fisher Scientific) and cultured in a humidified atmosphere at 37°C with 5% CO_2_. SH-SY5Y cells were purchased from ATCC, while Kelly and SK-N-AS cells were obtained from the European Collection of Authenticated Cell Cultures. Murine Ba/F3 cell line was obtained from RIKEN BioResource Center and maintained in RPMI 1640 medium supplemented with 10% FBS, penicillin/streptomycin and 10 ng/ml of recombinant mouse interleukin-3 (IL-3, Fujifilm-Wako Chemicals). *Mycoplasma* contamination was tested for using the Mycoplasma Detection Set (Takara Bio, Kusatsu, Japan).

### Electroporation and cell selection

Expression vectors of wild-type and F1174L-mutated *ALK* genes were kind gifts from Dr. J. Takita (Kyoto University, Kyoto, Japan). Ba/F3 cells were electroporated with the *ALK* vectors or pcDNA3 empty vector using a Neon transfection system (Thermo Fisher Scientific) at 1635 V/20 ms/1 pulse. Twenty-four hours after the electroporation, the cells were subjected to G418 selection (1 mg/ml, Merck) supplemented with IL-3 (10 ng/ml) for three weeks. Ectopic expression of the ALK proteins was confirmed by western blot.

### Cell proliferation assay (WST assay)

Neuroblastoma cell lines were seeded in 96-well plates at a final density of 3×10^3^ cells/well and allowed to attach overnight. The cells were then treated with DMSO or experimental compounds. After 72 h, cell proliferation was determined by WST assay using the Cell Counting Kit-8 (Fuji Film-Wako Chemicals) according to the manufacturer’s instructions and was quantified on an MTP-310 Microplate Reader (Corona Electric, Hitachinaka, Japan) or Multiskan Sky (Thermo Fisher Scientific). ALK-expressing Ba/F3 cells were seeded in 96-well plates at 5×10^3^ cells/well with different concentrations of IL-3 and cell viability was determined by WST assay. The cells were also treated with CCC-003 without IL-3 for 72 h and subjected to WST assay.

### Quantitative reverse transcriptase PCR (qRT-PCR)

Neuroblastoma cell lines were seeded in 6-well plates at a final density of 2.5×10^5^ cells/well and allowed to attach overnight. The cells were then treated with DMSO or experimental compounds. After 24 h, RNA was extracted using the RNeasy Plus Mini Kit (Qiagen, Hilden, Germany) following the manufacturer’s instructions. Purified RNA (300 ng–1 μg) was used for cDNA synthesis with SuperScript VILO Master Mix (Thermo Fisher Scientific), following the manufacturer’s protocol. The q-PCR Master Mix and specific primer sets were used for SYBR Green-based quantitative PCR (q-PCR; Applied Biosystems, Foster, CA, USA) on an Applied Biosystems StepOnePlus Real-Time PCR System (Thermo Fisher Scientific). The PCR primer sequences used were as follows: *ALK*, 5’-ACTCCAGGGAAGCATGG-3’ and 5’-CACTCGAAATGGGTTGTCTG-3’; *RPS18*, 5’-GAGGATGAGGTGGAACGTGT-3’ and 5’-TCTTCAGTCGCTCCAGGTCT-3’.

### Western blot

Neuroblastoma cell lines were seeded in a 10-cm dish at a final density of 1.5×10^6^ cells and allowed to attach overnight. The cells were then treated with DMSO or experimental compounds. After 48–72 h, cells were lysed in CHAPS lysis buffer with protease and phosphatase inhibitors (Roche, Basel, Switzerland), and 25 or 50 μg of total lysate were boiled for 5–10 min in CHAPS and sample buffer. Western blotting was performed using the following primary antibodies: anti-ALK (Cell Signaling Technology, Danvers, MA, USA), anti-Phospho-ALK (Cell Signaling Technology), anti-Shc (Cell Signaling Technology), anti-Phospho-Shc (Cell Signaling Technology), anti-PARP (Cell Signaling Technology), anti-cleaved Caspase-3 (Cell Signaling Technology), anti-GAPDH (Fuji Film-Wako Chemicals), and anti-Actin (Merck). Secondary antibodies used were as follows: HRP-linked anti-rabbit IgG (Cell Signaling Technology) and HRP-linked anti-mouse IgG (Cell Signaling Technology). The proteins were visualized using ImageQuant LAS 4000mini with enhanced chemiluminescence reagents (Thermo Fisher Scientific).

### Annexin Ⅴ staining

Neuroblastoma cells were exposed to experimental compounds for 48 h before the experiment. Annexin V staining was performed using the MEBCYTO Apoptosis Kit (MBL, Nagoya, Tokyo) according to the manufacturer’s instructions. Cells were analyzed on a BD FACSVerse flow cytometer using FlowJo Software (Becton Dickinson, Franklin Lakes, NJ, USA).

### *In vivo* study

SH-SY5Y cells (5×10^6^ cells) were mixed 1:1 with Matrigel (Corning, New York, NY, USA) and injected subcutaneously into the flanks of total 30 female BALB/c nu/nu mice (5–6 weeks old) purchased from Charles River Laboratories (Yokohama, Japan). After the tumor size reached 500 mm^3^, the mice were randomized into two groups and intravenously injected with DMSO or CCC-003 (0.3 mg/kg/week) once a week for 2 weeks (n = 8 in each group). Tumor volume (*W*×*W*×*L*/2) and body weights were measured every 3 or 4 days. Animal health was monitored by body weight and general assessment of animal behavioral activity. Adverse events were judged by body weight change and a humane endpoint was determined by 20% reduction in body weight. None of mice reached the endpoint during the experiments. When necessary, mice were anesthetized with 2% isoflurane. The mice were sacrificed when the tumor volume reached 2,000 mm^3^ by administration of anesthesia followed by cervical dislocation.

### Hematoxylin & eosin (H&E) and immunohistochemical staining

Compounds (0.3 mg/kg) or DMSO were injected intravenously into female BALB/c nu/nu mice harboring SH-SY5Y xenografts (n = 3 mice per group) when the tumor volume surpassed 500 mm^3^. After 24 h, tumors were resected and fixed in 4% paraformaldehyde. Sections (4 μm) were deparaffinized by immersion in xylene and were then rehydrated, followed by H&E staining according to standard procedures and immunostaining using an anti-cleaved caspase-3 antibody (Cell Signaling Technology). Cleaved caspase-3-positive cells were counted in 20 high-power fields of each tumor at ×400 magnification.

### Statistical analysis

Statistical analyses were performed using GraphPad Prism 6.0 (GraphPad Software, San Diego, CA, USA). For *in vitro* studies, one-way analysis of variance (ANOVA), followed by a Bonferroni the post-hoc test, was used for multiple group comparisons. For *in vivo* studies, two-way repeated measures ANOVA, followed by the Bonferroni post-hoc test, was used to evaluate differences in tumor volume and body weight between the groups. For cleaved caspase 3 immunostaining, unpaired *t*-test was used. *P*-values below 0.05 were considered statistically significant.

### Ethical statement

This study was performed in strict accordance with the recommendations in the Guide for the Care and Use of Laboratory Animals of the Ministry of Education, Culture, Sports, Science and Technology of Japan. The protocol was approved by the Committee on the Ethics of Animal Experiments of Chiba Cancer Center (IRB ID #18–2). All efforts were made to minimize their suffering.

## Results

### CCC-003 preferentially bound to the DNA sequence of mutant *ALK*

To disrupt the expression of the F1174L mutant *ALK* gene, we designed and synthesized PI polyamide CCC-003, which specifically recognizes the DNA sequence of mutated *ALK*, as well as a mismatch polyamide (Mismatch), which had no binding motif within the coding region of the *ALK* gene (Fig 1A, S1 and S2 Figs). CCC-003 possesses an alkylating moiety, *seco*-CBI, which can alkylate the adenine residue adjacent to a C to A mutation within the *ALK* gene (Fig 1B).

To validate the specific binding of CCC-003 to mutated *ALK*, we examined the affinity of PI polyamide to DNA fragments containing the F1174L-mutated sequence using an SPR assay. The non-alkylating variant CCC-003-Dp exhibited high affinity for target F1174L-mutated oligonucleotides when compared to oligonucleotides with the wild-type *ALK* sequence (Fig 1C and S3–S5 Figs). The dissociation constant (K_D_) values of CCC-003-Dp for the mutation and wild-type oligonucleotides were 6.37(± 0.41)×10^−8^ and 58.20(± 1.62)×10^−8^ M, respectively, and the binding affinity of CCC-003-Dp for the mutation sequence was 9.14-fold higher than for the wild-type sequence (Table 1). Preferential binding of PI polyamide to the mutation and wild-type sequence was not observed for the Mismatch.

**Table 1:** Binding affinities of PI polyamides for *ALK* mutation (F1174L) or *ALK* wild-type (WT) oligonucleotides.

PIP	*ALK* sequence	K_D_ [10^−8^ M]^a^	*k*_*a*_ [10^4^ M^-1^S^-1^]^b^	*k*_*d*_ [10^−3^ S^-1^]^c^	Specificity
CCC-003-Dp	F1174L	6.37 ± 0.41	1.97 ± 0.05	1.26 ± 0.02	9.14
Mismatch-Dp	F1174L	N.D.	N.D.	N.D.	N.D.
CCC-003-Dp	WT	58.20 ± 1.62	0.29 ± 0.021	1.71 ± 0.03	1.00
Mismatch-Dp	WT	N.D.	N.D.	N.D.	N.D.

^a)^Equilibrium dissociation constant

^b)^Association rate constant

^c)^Dissociation rate constant

N.D., not detected.

To determine the alkylation sites of target DNA fragments by CCC-003, we used high-resolution denaturing PAGE analysis. All DNA oligonucleotides used in this study are listed in Fig 1D. ODN3 and ODN4 were prepared as reference DNAs. As shown in Fig 1E, CCC-003 treatment demonstrated a fragment band corresponding to ODN3, indicating a specific alkylation by CCC-003 and heat-induced DNA cleavage at the targeted adenine adjacent to F1174L mutated sequence of ODN2. On the other hand, no obvious fragmented DNA was observed by Mismatch polyamide treatment. These data suggest that CCC-003 selectively alkylates the adenine nucleotide of F1174L-mutated *ALK* gene.

### CCC-003 suppressed cell viability and ALK expression in *ALK*-mutated neuroblastoma cells

**Fig 2:**
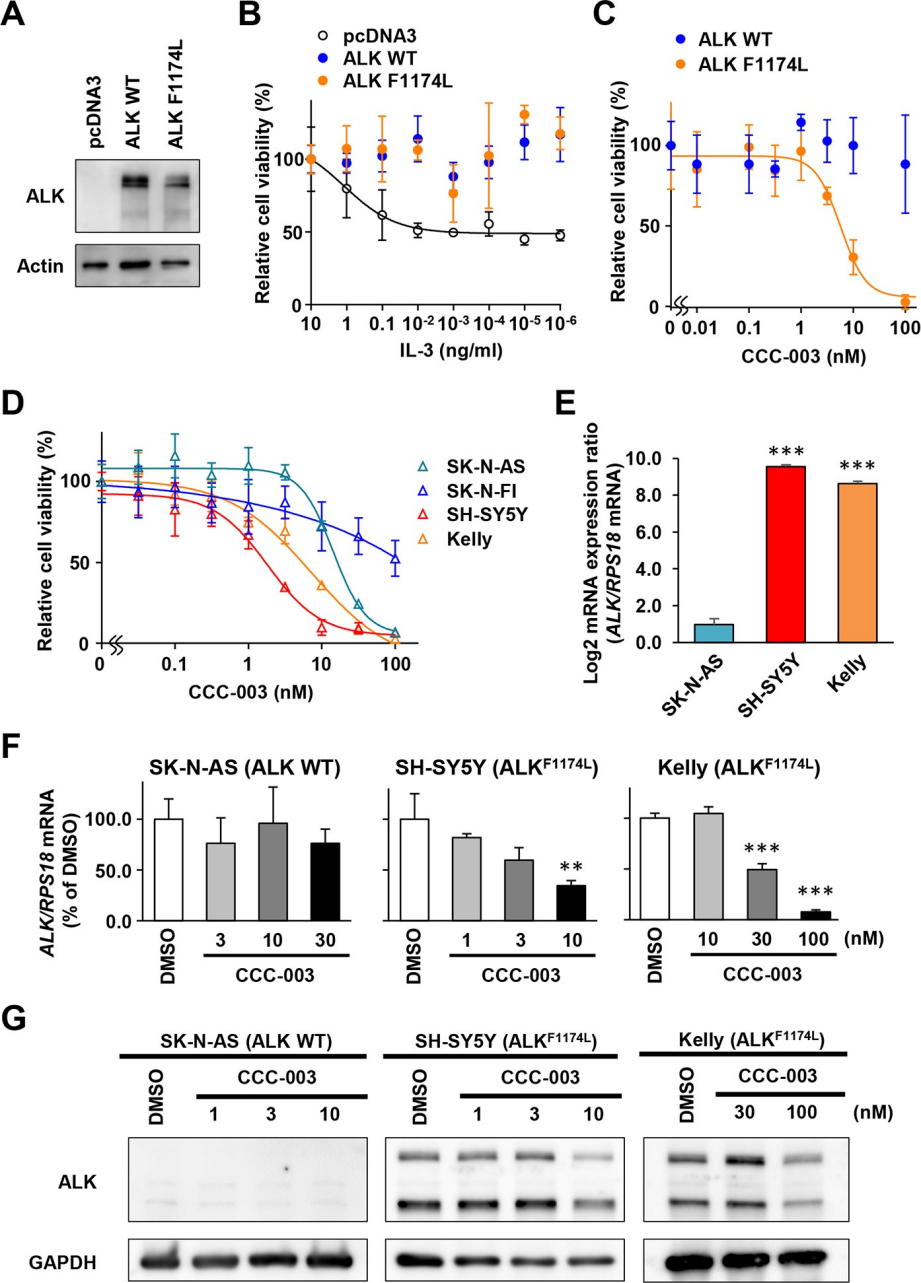
CCC-003 inhibits ALK expression in *ALK*-mutated neuroblastoma cell lines. (A) Western blotting analysis confirmed the expressions of wild-type (WT) and F1174L-mutated ALK in Ba/F3 cells after G418 selection. (B) WST assay in ALK-expressing Ba/F3 cells. The cells were cultured with the indicated concentrations of IL-3 for 72 h. (C) Ba/F3 cells expressing ALK were treated with 0.01–100 nM CCC-003 in the absence of IL-3 for 72 h and subjected to WST assay. (D) WST assay in neuroblastoma cells. SK-N-AS, SK-N-FI, SH-SY5Y, and Kelly cells were incubated with the indicated concentrations of CCC-003. 72 h after treatment, the percentage of viable cells was determined and depicted using GraphPad Prism. Error bars indicate the SD of data from triplicate experiments. (E) *ALK* wild-type SK-N-AS and *ALK* F1174L-mutated SH-SY5Y and Kelly cells were treated with DMSO for 24 h. *ALK* mRNA expression was analyzed using qRT-PCR. *RPS18* was used as an internal control. *P*-values were determined using one-way ANOVA followed by a Bonferroni post-hoc test (***, *P* < 0.001). Data are presented as the mean ± SD from three independent experiments. (F) SK-N-AS, SH-SY5Y, and Kelly cells were treated with 3–30, 1–10, or 10–100 nM CCC-003 for 24 h, respectively. *ALK* mRNA expression was analyzed using qRT-PCR. DMSO was used as a control treatment. *RPS18* expression was used as a housekeeping gene. *P*-values were determined using one-way ANOVA followed by a Bonferroni post-hoc test (**, *P* < 0.01; ***, *P* < 0.001). Data are presented as the mean ± SD from three independent experiments. (G) SK-N-AS and SH-SY5Y cells were treated with 1–10 nM CCC-003, and Kelly cells were treated with 30–100 nM CCC-003 for 48 h. ALK protein levels were analyzed using western blotting.

We assessed anti-proliferative activity of CCC-003 on *ALK* F1174L-mutant cells using IL-3-dependent murine hematopoietic cell line, Ba/F3. The cells were electroporated with expression vectors for wild-type and F1174L-mutated *ALK* or empty pcDNA3 vector (Fig 2A). G418 selected cells that expressed ALK proteins showed sustained viability after IL-3 removal (Fig 2B). We treated these ALK-expressing Ba/F3 cells with CCC-003 and found that CCC-003 suppressed IL-3-independent cell viability in the cells with F1174L-mutated ALK expression but not in those expressing wild-type ALK (Fig 2C). We next performed WST assays in human neuroblastoma cells to examine the effect of CCC-003 on cell proliferation. The IC_50_ values of CCC-003 in *ALK*-mutated SH-SY5Y and Kelly cells (1.8 and 7.3 nM) were lower than those for wild-type *ALK*-harboring SK-N-AS and SK-N-FI cells (14.3 and >100 nM, respectively, Fig 2D). The expression level of *ALK* in neuroblastoma cell lines was examined by qRT-PCR. SH-SY5Y and Kelly cells expressed F1174L-mutated *ALK*, while SK-N-AS cells expressed wild-type *ALK* at low levels (Fig 2E). In *ALK*-mutated neuroblastoma cells, CCC-003 significantly reduced the mRNA and protein expression levels of ALK in a dose-dependent manner (Fig 2F and 2G). These results suggest that CCC-003 downregulates F1174L-mutated *ALK* expression and suppresses the proliferation of neuroblastoma cells.

### CCC-003 affected the F1174L-mutated *ALK* gene in a different manner from ALK inhibitors

**Fig 3:**
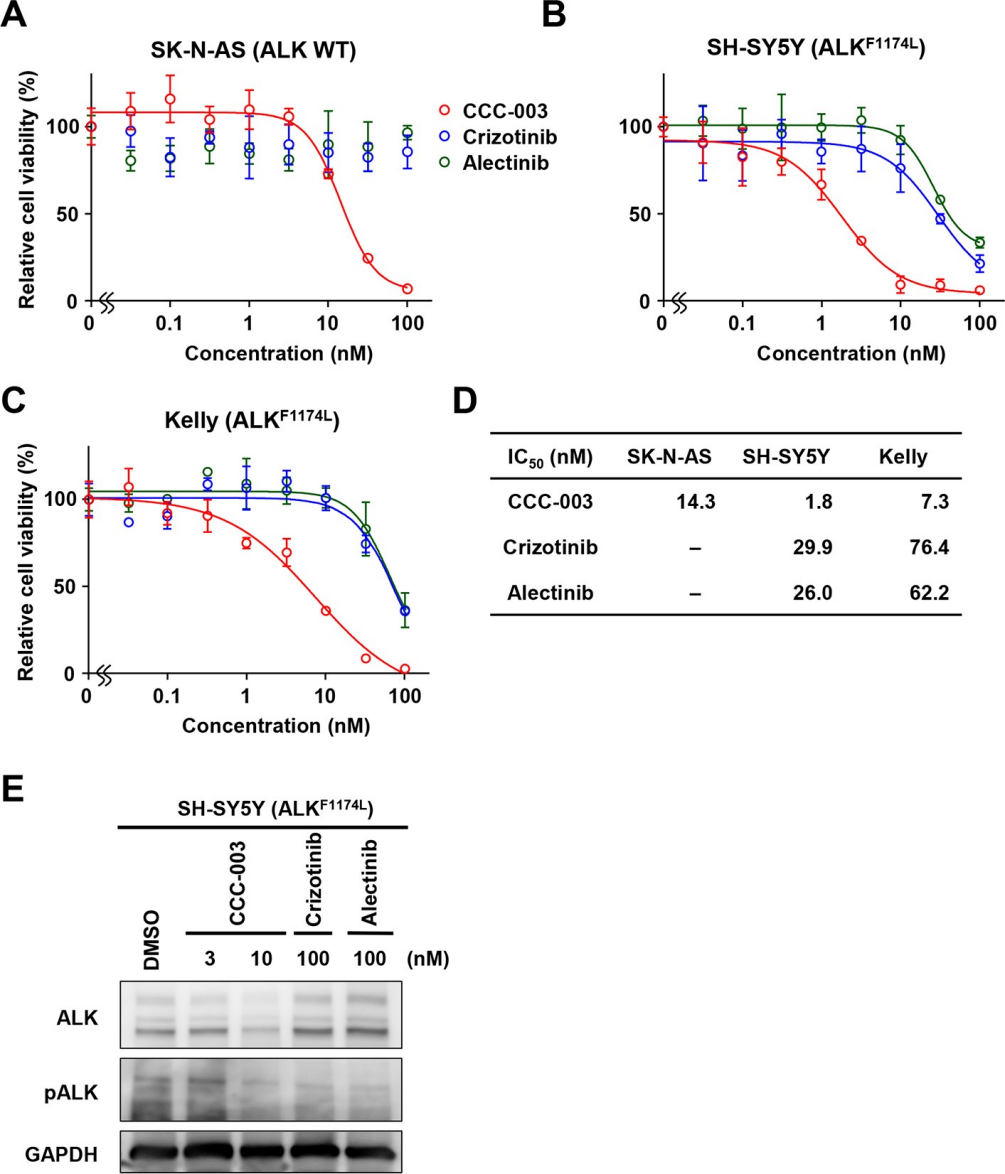
CCC-003 inhibits cell proliferation and ALK expression in *ALK*-mutated neuroblastoma cell lines to a greater extent than other ALK inhibitors. (A, B, C) WST assay in *ALK* wild-type and *ALK*-mutated cells. (A) SK-N-AS cells were incubated for 72 h after treatment with CCC-003 or ALK inhibitors crizotinib and alectinib. (B) SH-SY5Y cells were incubated for 72 h after treatment with CCC-003 or ALK inhibitors. (C) Kelly cells were incubated for 72 h after treatment with CCC-003 or ALK inhibitors. (D) IC_50_ values of CCC-003, crizotinib, and alectinib. (E) SH-SY5Y cells were treated with 3–10 nM CCC-003 or 100 nM ALK inhibitors for 72 h. ALK and pALK protein levels were determined using western blotting analysis.

We compared CCC-003 to two ALK inhibitors, crizotinib and alectinib. The WST assay demonstrated that cell growth inhibition in *ALK*-mutated cells by CCC-003 was more pronounced when compared to crizotinib and alectinib (Fig 3A–3D). As expected, ALK inhibitors suppressed the phosphorylated form of ALK without changing ALK protein expression in *ALK*-mutated cells (Fig 3E). In contrast, CCC-003 treatment suppressed both the phosphorylated form and total ALK expression. These data suggest that CCC-003 inhibits ALK activation by suppressing its gene expression through a mode of action that is different from that of other small-molecule ALK inhibitors.

### *ALK*-mutated neuroblastoma cells were more sensitive to CCC-003 than to the mismatch polyamide

**Fig 4:**
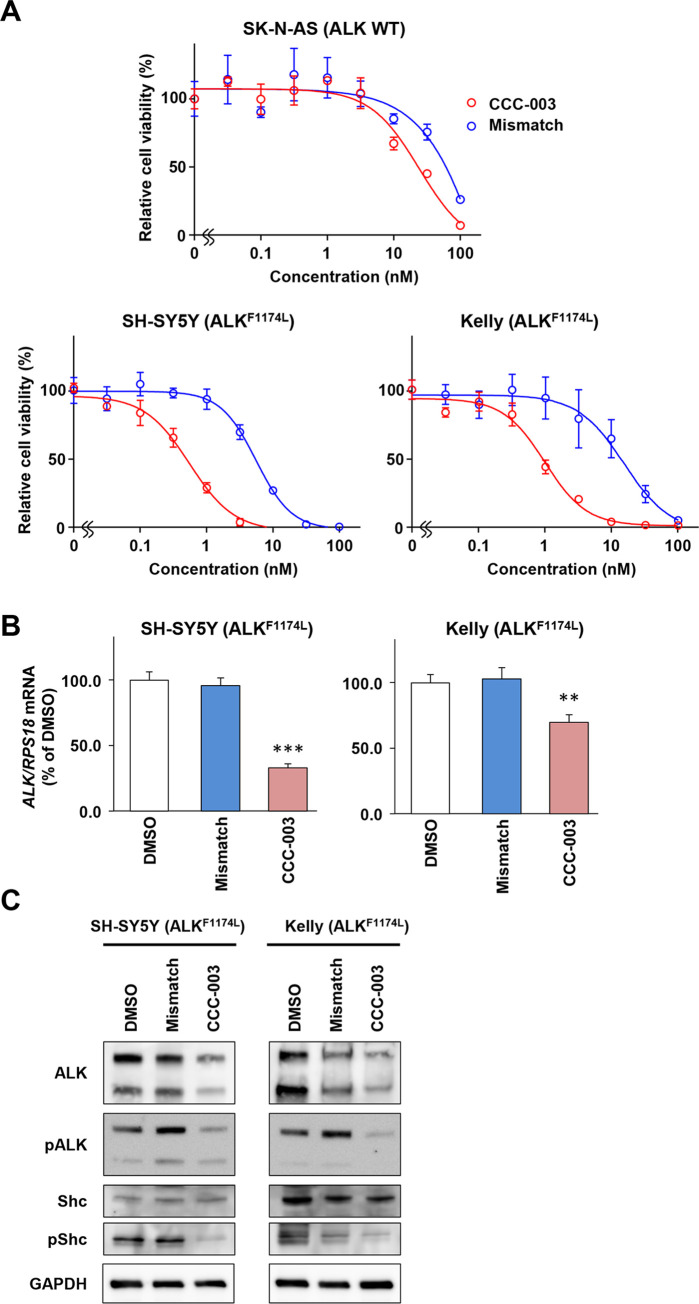
CCC-003 specifically suppresses ALK expression in *ALK*-mutated neuroblastoma cell lines. (A) WST assay in *ALK* wild-type and *ALK*-mutated cells. SK-N-AS, SH-SY5Y and Kelly cells were incubated for 72 h after treatment with CCC-003 or mismatch polyamide. (B) SH-SY5Y cells were treated with 3 nM CCC-003 or mismatch polyamide, and Kelly cells were treated with 30 nM CCC-003 or mismatch polyamide for 24 h. *ALK* mRNA expression was analyzed using qRT-PCR. *P*-values were determined using one-way ANOVA followed by a Bonferroni post-hoc test (**, *P* < 0.01; ***, *P* < 0.001). (C) SH-SY5Y cells were treated with 3 nM CCC-003, and Kelly cells were treated with 30 nM CCC-003 for 48 h. ALK, pALK, Shc, and pShc protein levels were determined using western blotting.

To further assess the targeting specificity, we compared CCC-003 with Mismatch polyamide in WST assays. Mismatch polyamide demonstrated 10.0- and 15.6-fold higher IC_50_ values than CCC-003 in SH-SY5Y and Kelly cells, respectively. In contrast, a comparable IC_50_ value of Mismatch polyamide was observed in SK-N-AS cells (68.8 nM; Fig 4A). qRT-PCR and western blotting analyses revealed that the mismatch polyamide failed to suppress the expression of *ALK* at both the mRNA and protein levels (Fig 4B and 4C). Further, western blot analyses revealed the downregulation of Shc adaptor protein phosphorylation.

### CCC-003 induced apoptotic cell death in *ALK*-mutated neuroblastoma cells

**Fig 5:**
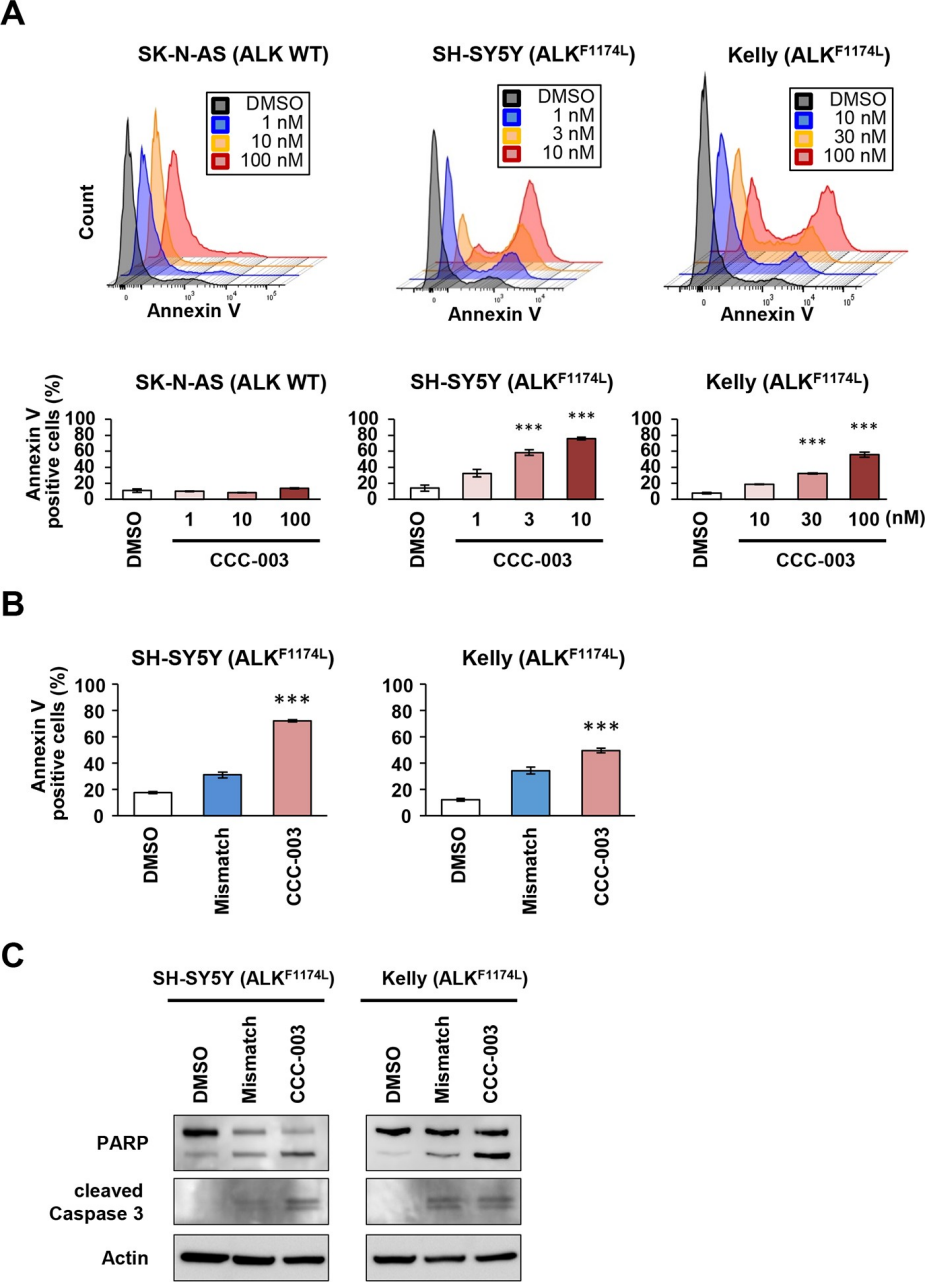
CCC-003 induces apoptotic cell death in *ALK*-mutated neuroblastoma cell lines. (A, B) Flow cytometric analysis by Annexin V staining of neuroblastoma cells. *P*-values were determined using one-way ANOVA followed by a Bonferroni post-hoc test (***, *P* < 0.001). (A) SK-N-AS, SH-SY5Y, and Kelly cells were treated with 1–100, 1–10, and 10–100 nM CCC-003 for 48 h, respectively. Representative images of Annexin V-positive cells detected using flow cytometry. (B) SH-SY5Y cells were treated with 3 nM CCC-003 or mismatch polyamide, and Kelly cells were treated with 30 nM CCC-003 or mismatch polyamide for 48 h. (C) SH-SY5Y cells were treated with 3 nM CCC-003 or mismatch polyamide, and Kelly cells were treated with 30 nM CCC-003 or mismatch polyamide for 48 h. Protein expression of cleaved PARP and cleaved caspase-3 was determined using western blotting.

To clarify whether CCC-003 induces cell death in *ALK*-mutated neuroblastoma cells, we performed Annexin V staining. At 48 h after treatment, CCC-003 significantly increased the number of Annexin V-positive SH-SY5Y and Kelly cells in a dose-dependent manner, while the mismatch polyamide had little influence on the viability of *ALK*-mutated neuroblastoma cells (Fig 5A and 5B and S6 Fig). The number of Annexin V-positive SK-N-AS cells did not change after CCC-003 treatment (Fig 5A). Western blotting analyses revealed that the levels of apoptotic markers cleaved PARP and cleaved caspase-3 were increased in SH-SY5Y and Kelly cells (Fig 5C).

### CCC-003 suppressed tumor progression in an SH-SY5Y-xenograft mouse model

**Fig 6:**
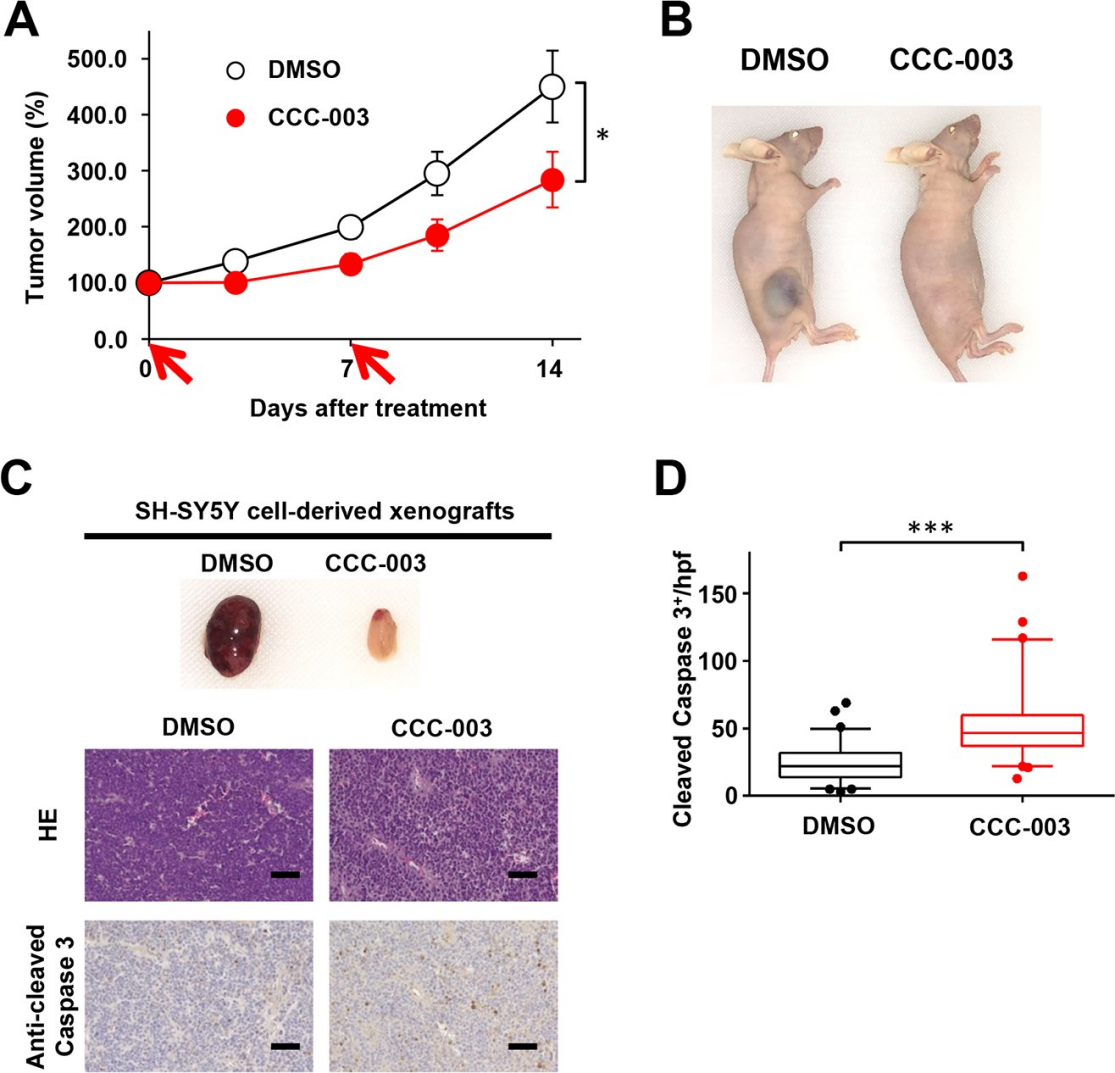
CCC-003 suppresses tumor growth in human *ALK*-mutated neuroblastoma xenograft models. (A, B, C) SH-SY5Y cells were subcutaneously injected into the flanks of female immune-deficient BALB/c nu/nu mice. Administration of CCC-003 began when the average tumor size reached 500 mm^3^. (A) The tumor volume of mice was measured twice a week. Red arrows indicate the timepoints of CCC-003 administration. *P*-values were determined using repeated measures ANOVA followed by a Bonferroni post-hoc test (*, *P* < 0.05). Data are presented as the mean ± SD. DMSO was used as a control. (B) Representative images of mice with SH-SY5Y xenografts at 24 h after administration. (C) Representative images of SH-SY5Y xenograft tumors at 24 h after administration. At 24 h after administration, the tumor tissues were collected and used for immunohistochemistry with HE and an anti-cleaved caspase-3 antibody. Scale bars, 50 μm. (D) The number of cleaved caspase-3-positive cells was determined in tumor tissues. *P-*values were determined using a paired *t*-test (***, *P* < 0.001).

We evaluated the anti-tumor potential of CCC-003 in a neuroblastoma xenograft model established using SH-SY5Y cells. Tumor-bearing mice that received an intravenous injection of CCC-003 at 0.3 mg/kg BW displayed significant suppression of tumor growth compared with vehicle-treated mice, without weight loss (Fig 6A and S7A Fig). We also performed histological analysis of SH-SY5Y xenograft-derived tumor tissue sections 24 h after administration of CCC-003 (Fig 6B and 6C). HE staining revealed chromatin condensation and nuclear fragmentation in tumors treated with CCC-003 (Fig 6C). Immunohistochemical staining using the cleaved caspase-3 antibody revealed an elevated level of apoptotic cell death in tumor tissues after CCC-003 administration (Fig 6C and 6D). There were no histological changes in tissues of the lung, liver, and kidney after CCC-003 treatment, suggestive of minimal side effects in mice (S7B Fig).

## Discussion

ALK TKIs have been evaluated in ALK-driven neuroblastoma cell lines, mouse models, and ongoing clinical trials [2, 21, 22]. Currently, alectinib and crizotinib are considered effective in the treatment of ALK-positive lung cancer [23]. However, relapse due to acquired ALK inhibitor resistance caused by secondary *ALK* mutations often occurs in lung cancer patients [24, 25]. A similar resistance development event may occur in neuroblastoma patients after ALK inhibitor treatment. To overcome the issue of ALK inhibitor resistance in neuroblastoma, we used a novel PI polyamide designed to target the mutant *ALK* gene in a manner different from that of ATP-competitive inhibitors. *ALK* F1174L mutation is a major aberration observed in both primary and relapsed neuroblastomas [2, 26]. Herein, we developed CCC-003, an alkylating PI polyamide, which preferentially binds the genomic DNA of the *ALK* F1174L mutation, suppressing its expression and retarding the growth of *ALK* F1174L-positive xenograft tumors. F1174L-mutated ALK expression was previously demonstrated to have an anti-apoptotic effect during neuroblastoma genesis [27, 28]. CCC-003 treatment upregulated apoptotic cell death in neuroblastoma cells and tumor tissues, suggesting that the suppression of oncogenic mutated *ALK* by CCC-003 was sufficient to upregulate pro-apoptotic signaling and thus exert anti-tumor effects in ALK-positive neuroblastoma.

In accordance with previous studies [29, 30], treatment with ALK inhibitors suppressed the phosphorylated form of ALK without affecting total ALK levels in SH-SY5Y and Kelly cells. In contrast, CCC-003 treatment downregulated ALK expression and resulted in a reduced level of its phosphorylated form, suggesting a distinct mode of action for CCC-003 compared to other small molecule TKIs. According to previous reports [17, 31, 32], covalent alkylation of the coding DNA strand by PI polyamide with *seco*-CBI specifically inhibits the transcription of target genes. In the case of CCC-003, the expression of *ALK* mRNA was suppressed at 24 h after treatment, suggesting that CCC-003 rapidly translocated into the cell nucleus, and selective alkylation occurred at the mutation site of *ALK*. ALK expression was slightly reduced by mismatch polyamide treatment. The mismatch polyamide was designed to have no binding sequence in the region of *ALK* and thus exhibited low affinity to the wild-type sequence oligonucleotides in SPR assays, indicative of the sequence-specific manner of *ALK* inhibition by CCC-003. Even though CCC-003 has the unique property of a gene silencer, potential off-target effects on other functional genes are a concern because CCC-003 recognizes a relatively short DNA motif of 10 bp. Further examination of CCC-003 binding sites in the human genome is necessary to clarify its genome-wide impact and possible side effects in neuroblastoma patients.

Alectinib has been shown to have high efficacy against crizotinib-resistant F1174L *ALK*-positive cancer cells [19, 33, 34]. Nevertheless, grade 3–4 adverse events are observed in response to alectinib in *ALK*-rearranged lung cancer [33, 35]. In the present study, CCC-003 had a 10-fold lower IC_50_ value compared to these two inhibitors, suggesting that it is more cytotoxic in neuroblastoma cells than other ALK inhibitors. CCC-003 treatment resulted in decreased phosphorylation of the Shc adaptor protein, indicating that proliferative signaling via ALK may be downregulated by CCC-003 at a dose low enough to avoid possible side effects. Of note, intratumoral accumulation has been reported for the *in vivo* dynamics of PI polyamides [36, 37]. These unique characteristics of PI polyamides may have led to the negligible adverse effects observed in the xenograft mouse models treated with CCC-003.

Comparable IC_50_ values for alectinib were observed among neuroblastoma cell lines, regardless of the *MYCN* amplification status of cells [34]. In the present study, when compared to SH-SY5Y cells, CCC-003 exhibited weaker cytotoxic activity with a higher IC_50_ value in Kelly cells. There are several possible reasons for the different sensitivity of these two cell lines. According to a previous report, MYCN expression determines anti-cancer drug sensitivity in neuroblastoma cells [38]. Our results indicated that the amplified *MYCN* in Kelly cells might affect their sensitivity to CCC-003. Moreover, a study reports that the sensitivity to apoptosis of MYCN-overexpressing neuroblastomas is increased through the DNA damage response [39]. To clarify the association between *MYCN* status and sensitivity to CCC-003 in neuroblastoma cells, further examination of the DNA damage repair system in SH-SY5Y and Kelly cells would be necessary.

In the present study, we successfully developed CCC-003, a novel PI polyamide conjugated with a DNA-alkylating agent. CCC-003 exhibited high cytotoxicity in *ALK*-mutated neuroblastoma cells. The current work highlighted a novel strategy for the development of therapeutic agents for neuroblastoma capable of direct alkylation of target mutant gene loci. The specific binding to mutated DNA is a unique mode of action of PI polyamide leading to the induction of tumor cell death. Owing to its specific binding, CCC-003 could be a novel therapeutic agent for ALK-positive neuroblastoma without significant side effects. Since PI polyamides can be designed to recognize and target the sequences of aberrant genes, these unique compounds could serve as novel personalized agents against different types of malignant tumors harboring various genetic aberrations, including point mutations, translocations, as well as amplifications.

## Supporting information

S1 Fig HPLC analysis and LC-MS spectrum identification of CCC-003. (A) HPLC of isolated CCC-003. (B) LC-MS spectrum of isolated CCC-003. LC-MS m/z calculated for C 102, H 105, Cl 1, N 34, O 18, [M+2H]^2+^ 1065.40; found 1065.85, [M+3H]^3+^ 710.6; found 710.95.
(TIF)

S2 Fig HPLC analysis and LC-MS spectrum identification of the mismatch. (A) HPLC of isolated Mismatch. (B) LC-MS spectrum of isolated Mismatch. LC-MS m/z calculated for C 101, H 104, Cl 1, N 35, O 18, [M+2H]^2+^ 1065.40; found 1065.90, [M+3H]^3+^ 710.93; found 711.15.
(TIF)

S3 Fig HPLC analysis and LC-MS spectrum identification of CCC-003-Dp. (A) Structure of CCC-003-Dp. (B) HPLC of isolated CCC-003-Dp. (C) LC-MS spectrum of isolated CCC-003-Dp. LC-MS m/z calculated for C 94, H 107, N 35, O 17, [M+H]^+^ 1997.86; found 1996.85, [M+2H]^2+^ 999.93; found 1000.30, [M+3H]^3+^ 667.00; found 667.20.
(TIF)

S4 Fig HPLC analysis and LC-MS spectrum identification of Mismatch-Dp. (A) Structure of Mismatch-Dp. (B) HPLC of isolated Mismatch-Dp. (C) LC-MS spectrum of isolated Mismatch-Dp. LC-MS m/z calculated for C 93, H 106, N 36, O 17, [M+H]^+^ 1997.86; found 1996.60, [M+2H]^2+^ 1000.43; found 1000.35, [M+3H]^3+^ 667.28; found 667.25.
(TIF)

S5 Fig Schematic drawing of CCC-003-Dp and Mismatch-Dp at sites of double-stranded DNA with *ALK* F1174L mutation or *ALK* wild-type sequences. Blue open circles represent pyrrole moieties, and red ones represent imidazole. (A) CCC-003-Dp at the sites of double-stranded DNA with the *ALK* F1174L mutation sequence. (B) Mismatch-Dp at the sites of double-stranded DNA with the *ALK* F1174L mutation sequence. (C) CCC-003-Dp at sites of double-stranded DNA with the *ALK* wild-type sequence. (D) Mismatch-Dp at the sites of double-stranded DNA with the *ALK* wild-type sequence.
(TIF)

S6 Fig Representative images of Annexin V-positive cells among *ALK*-mutated cells. SH-SY5Y cells were treated with 3 nM CCC-003 or mismatch polyamide, and Kelly cells were treated with 30 nM CCC-003 or mismatch polyamide for 48 h. Representative images show Annexin V-positive cells detected using flow cytometry.
(TIF)

S7 Fig Body weight and organ histology of mice after administration of CCC-003 in human *ALK*-mutated neuroblastoma xenograft models. SH-SY5Y cells were subcutaneously injected into the flanks of female immune-deficient BALB/c nu/nu mice. Administration of CCC-003 began when the average tumor size reached 500 mm^3^. (A) Body weight of mice was measured twice a week. Red arrows, timepoint of CCC-003 administration. Data are represented as the mean ± SD. DMSO was used as a control. (B) After reaching the end point, tissues of the lung, liver, and kidney were collected and used for HE staining. Scale bars, 50 μm.
(TIF)

S1 Dataset (XLSX)

S1 Raw images (PDF)
