# Supplementary figures and images for: Targeting anaplastic lymphoma kinase (ALK) gene alterations in neuroblastoma by using alkylating pyrrole-imidazole polyamides
Source: PLoS One. 2021 Sep 30;16(9):e0257718. doi: 10.1371/journal.pone.0257718 (PMC8483358; doi:10.1371/journal.pone.0257718)

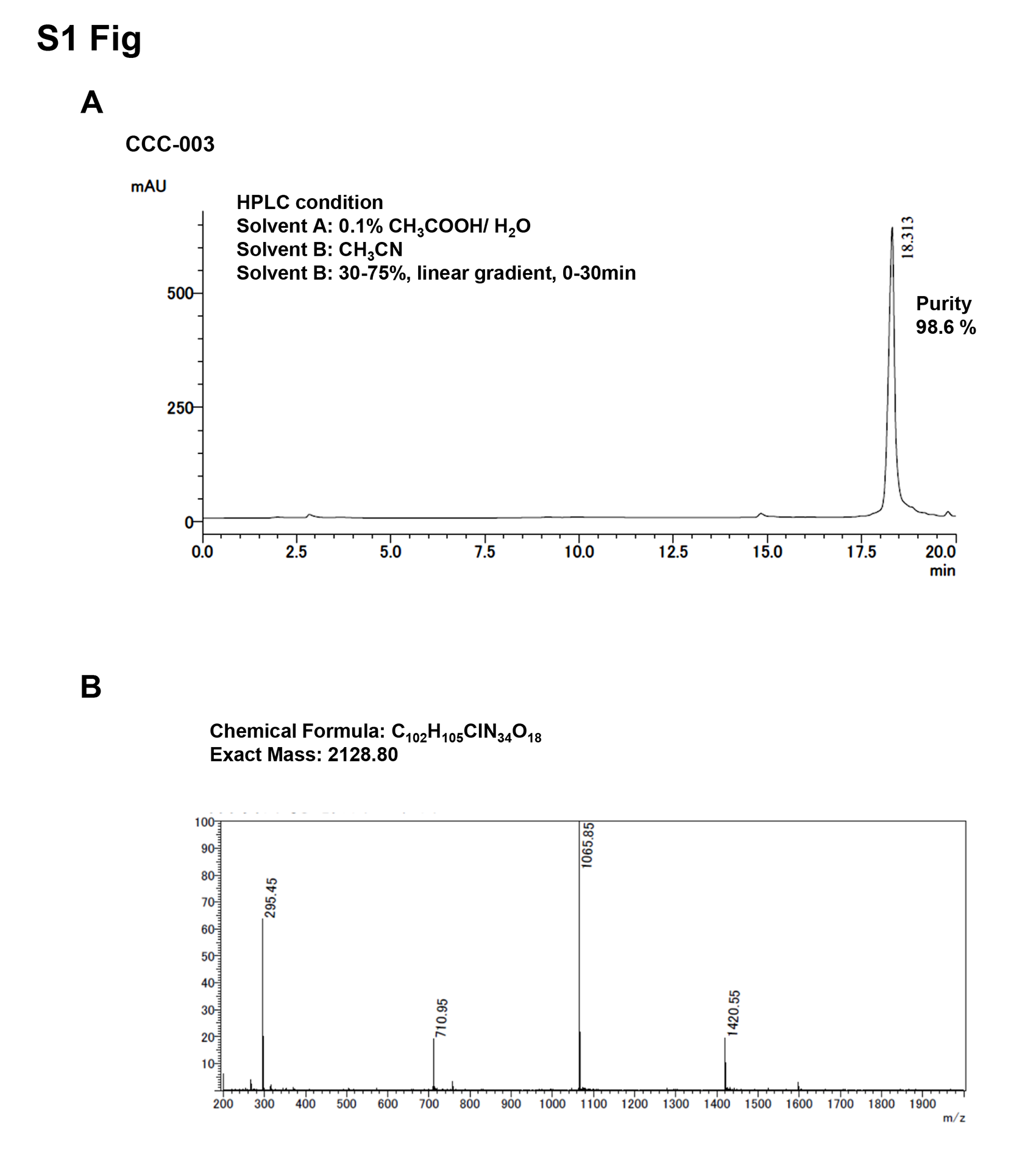

Supplement: S1 Fig — (A) HPLC of isolated CCC-003. (B) LC-MS spectrum of isolated CCC-003. LC-MS m/z calculated for C 102, H 105, Cl 1, N 34, O 18, [M+2H]2+ 1065.40; found 1065.85, [M+3H]3+ 710.6; found 710.95. (TIF) [file pone.0257718.s001.tif]

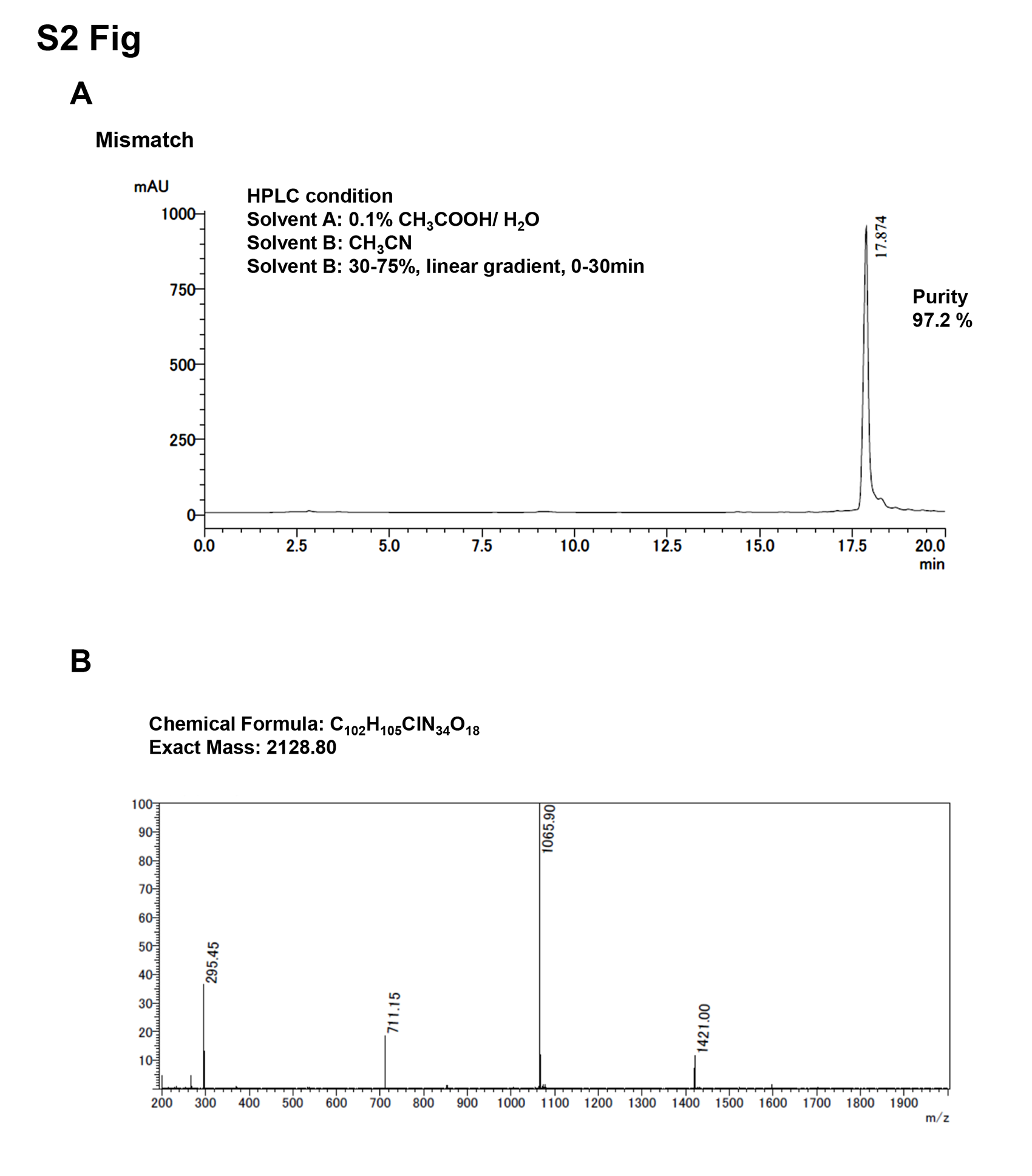

Supplement: S2 Fig — (A) HPLC of isolated Mismatch. (B) LC-MS spectrum of isolated Mismatch. LC-MS m/z calculated for C 101, H 104, Cl 1, N 35, O 18, [M+2H]2+ 1065.40; found 1065.90, [M+3H]3+ 710.93; found 711.15. (TIF) [file pone.0257718.s002.tif]

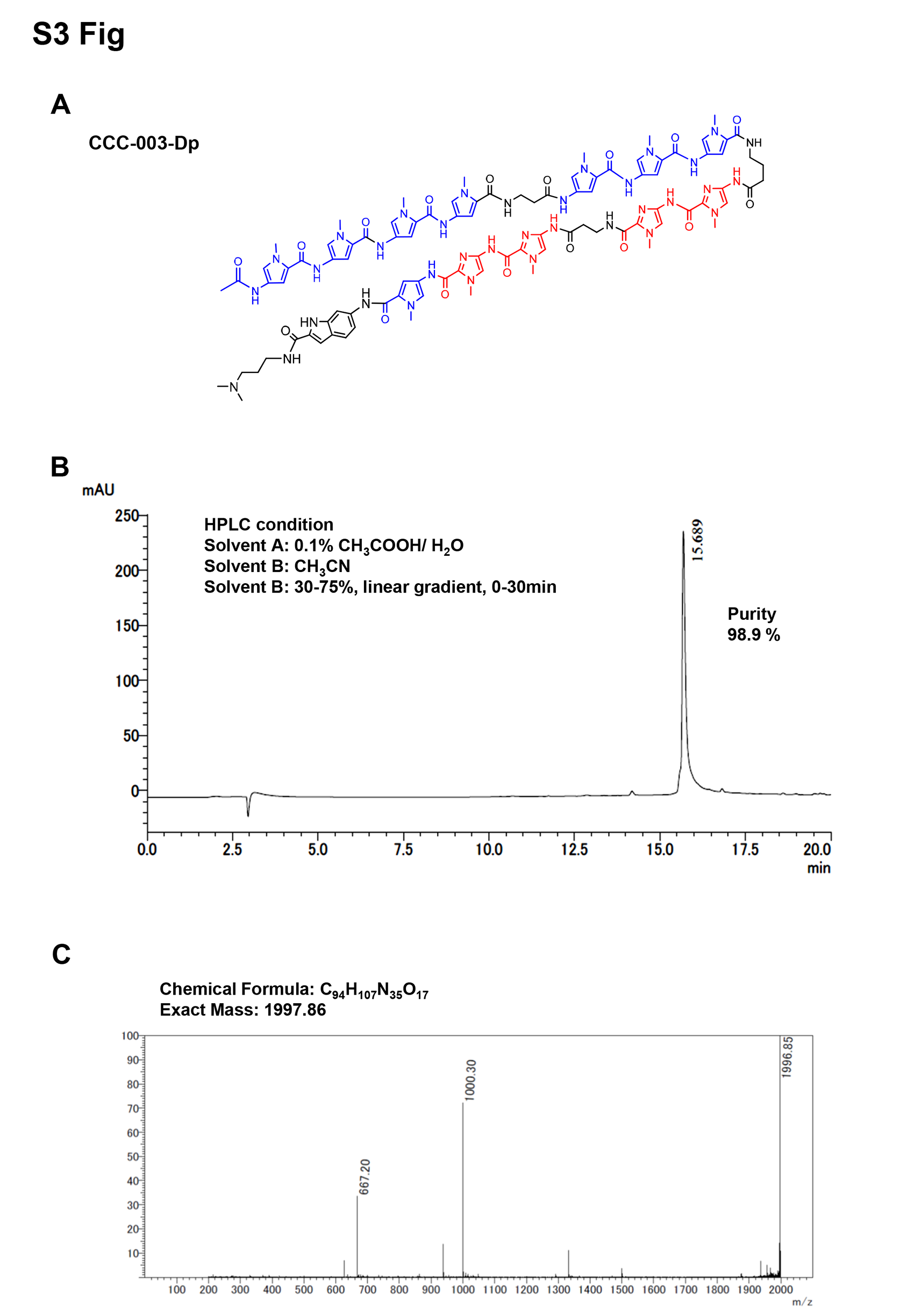

Supplement: S3 Fig — (A) Structure of CCC-003-Dp. (B) HPLC of isolated CCC-003-Dp. (C) LC-MS spectrum of isolated CCC-003-Dp. LC-MS m/z calculated for C 94, H 107, N 35, O 17, [M+H]+ 1997.86; found 1996.85, [M+2H]2+ 999.93; found 1000.30, [M+3H]3+ 667.00; found 667.20. (TIF) [file pone.0257718.s003.tif]

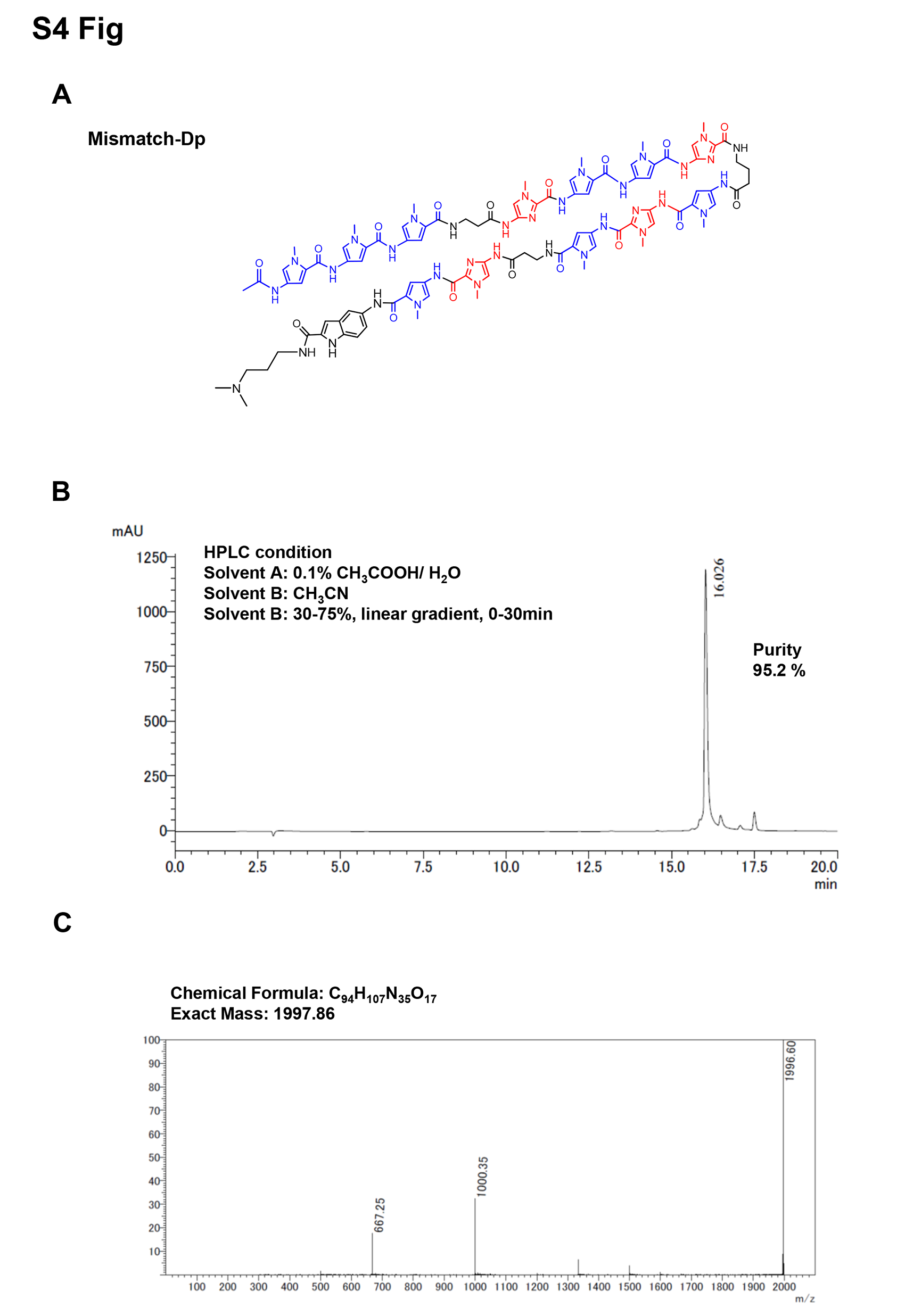

Supplement: S4 Fig — (A) Structure of Mismatch-Dp. (B) HPLC of isolated Mismatch-Dp. (C) LC-MS spectrum of isolated Mismatch-Dp. LC-MS m/z calculated for C 93, H 106, N 36, O 17, [M+H]+ 1997.86; found 1996.60, [M+2H]2+ 1000.43; found 1000.35, [M+3H]3+ 667.28; found 667.25. (TIF) [file pone.0257718.s004.tif]

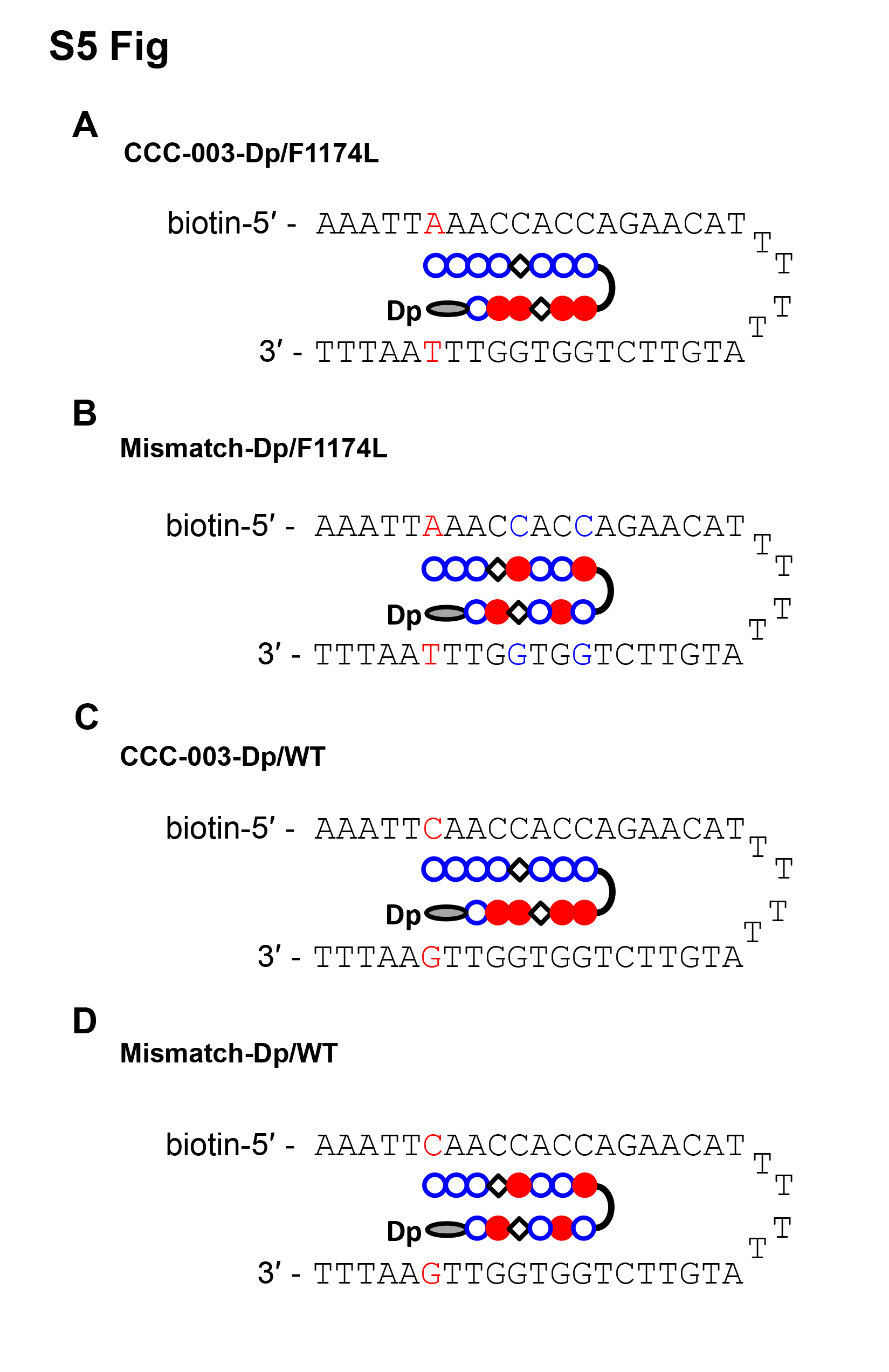

Supplement: S5 Fig — Blue open circles represent pyrrole moieties, and red ones represent imidazole. (A) CCC-003-Dp at the sites of double-stranded DNA with the ALK F1174L mutation sequence. (B) Mismatch-Dp at the sites of double-stranded DNA with the ALK F1174L mutation sequence. (C) CCC-003-Dp at sites of double-stranded DNA with the ALK wild-type sequence. (D) Mismatch-Dp at the sites of double-stranded DNA with the ALK wild-type sequence. (TIF) [file pone.0257718.s005.tif]

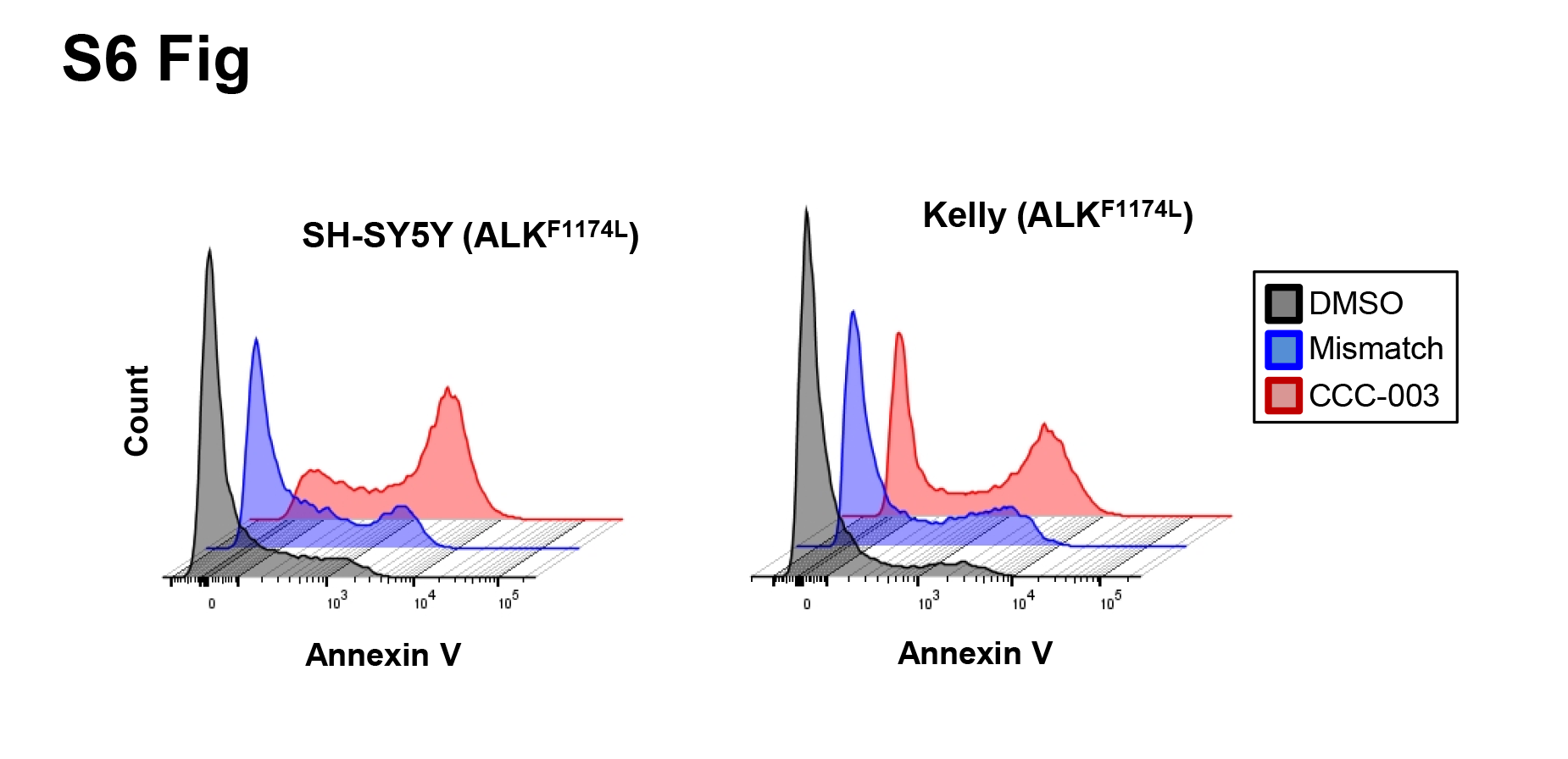

Supplement: S6 Fig — SH-SY5Y cells were treated with 3 nM CCC-003 or mismatch polyamide, and Kelly cells were treated with 30 nM CCC-003 or mismatch polyamide for 48 h. Representative images show Annexin V-positive cells detected using flow cytometry. (TIF) [file pone.0257718.s006.tif]

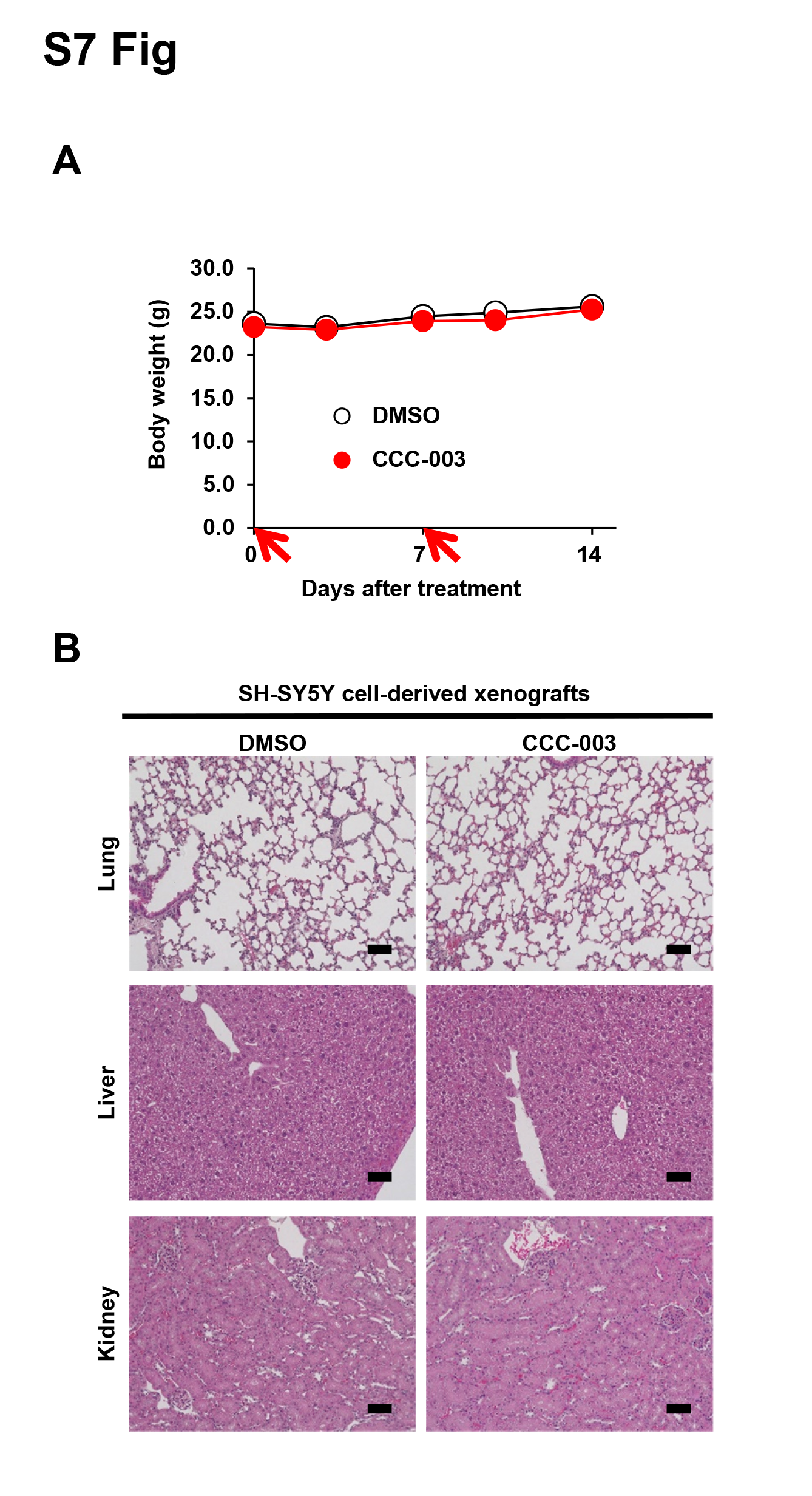

Supplement: S7 Fig — SH-SY5Y cells were subcutaneously injected into the flanks of female immune-deficient BALB/c nu/nu mice. Administration of CCC-003 began when the average tumor size reached 500 mm3. (A) Body weight of mice was measured twice a week. Red arrows, timepoint of CCC-003 administration. Data are represented as the mean ± SD. DMSO was used as a control. (B) After reaching the end point, tissues of the lung, liver, and kidney were collected and used for HE staining. Scale bars, 50 μm. (TIF) [file pone.0257718.s007.tif]
