# Supplementary material for: Targeting anaplastic lymphoma kinase (ALK) gene alterations in neuroblastoma by using alkylating pyrrole-imidazole polyamides
Source: PLoS One. 2021 Sep 30;16(9):e0257718. doi: 10.1371/journal.pone.0257718 (PMC8483358; doi:10.1371/journal.pone.0257718)
Supplement: S1 Raw images — (PDF) [file pone.0257718.s009.pdf]

Ota et al.

Fig 1E

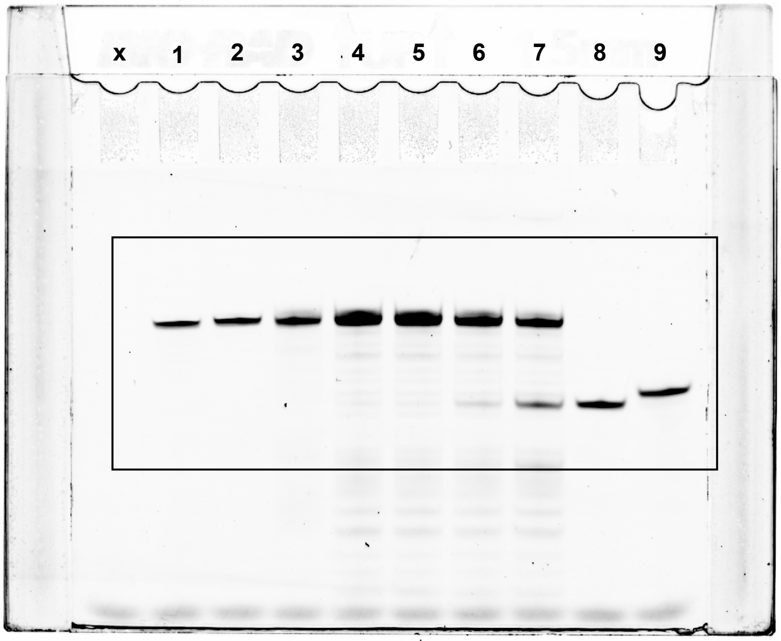

1: ODN1
2: ODN2
3: dsDNA
4: dsDNA + DMSO
5: dsDNA + 5.0 μM Mismatch
6: dsDNA + 1.0 μM CCC-003
7: dsDNA + 5.0 μM CCC-003
8: ODN3 (15mer)
9: ODN4 (16mer)

Fig 2A

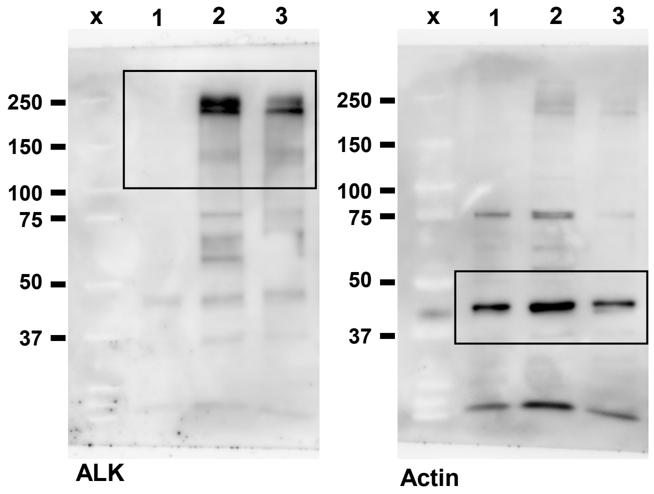

1. pcDNA3
2. ALK WT
3. ALK F1174L

Fig 2G

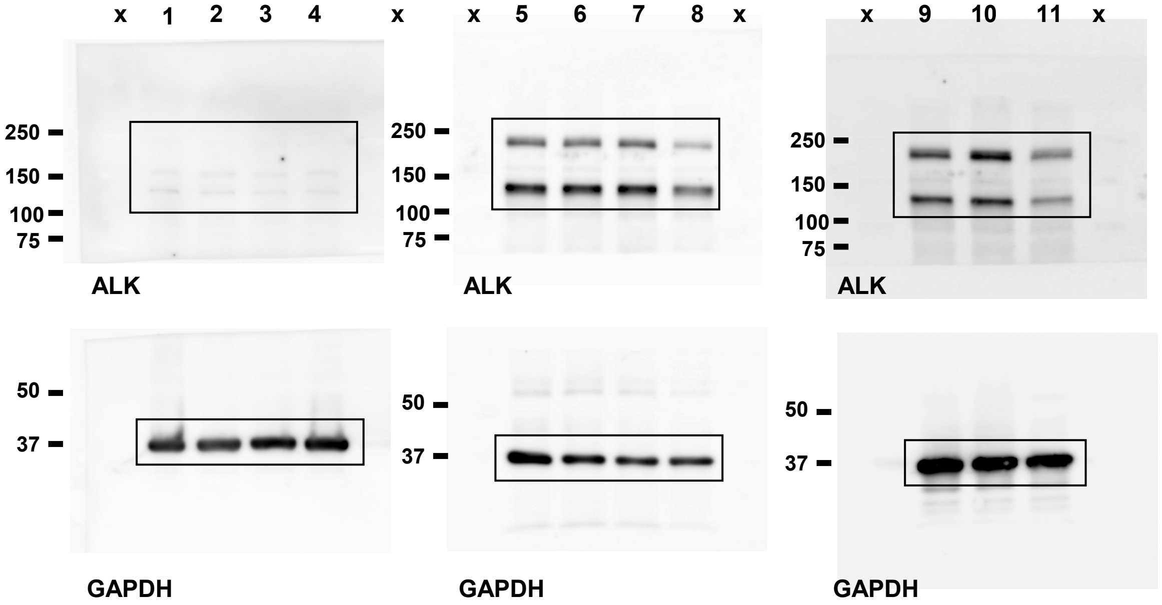

1. SK-N-AS; DMSO
2. SK-N-AS; CCC-003, 1 nM
3. SK-N-AS; CCC-003, 3 nM
4. SK-N-AS; CCC-003, 10 nM

5. SH-SY5Y; DMSO
6. SH-SY5Y; CCC-003, 1 nM
7. SH-SY5Y; CCC-003, 3 nM
8. SH-SY5Y; CCC-003, 10 nM

9. Kelly; DMSO
10. Kelly; CCC-003, 30 nM
11. Kelly; CCC-003, 100 nM

Fig 3E

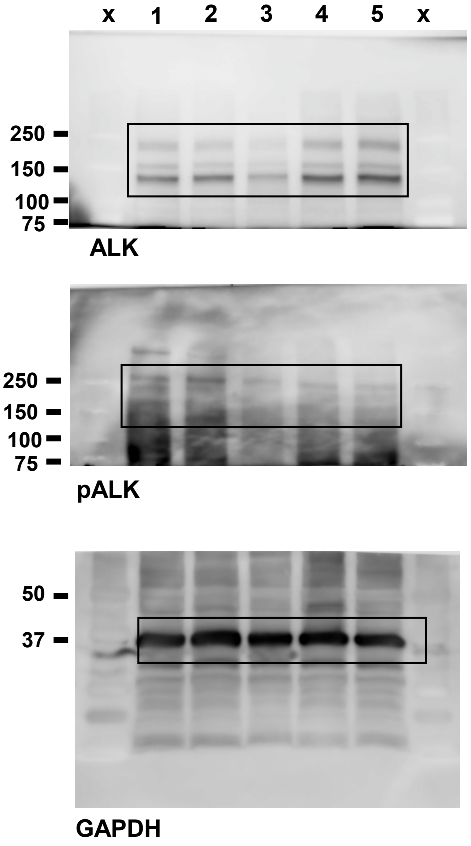

1. SH-SY5Y; DMSO
2. SH-SY5Y; CCC-003, 3 nM
3. SH-SY5Y; CCC-003, 10 nM
4. SH-SY5Y; Crizotinib, 100 nM
5. SH-SY5Y; Alectinib, 100 nM

Fig 4C

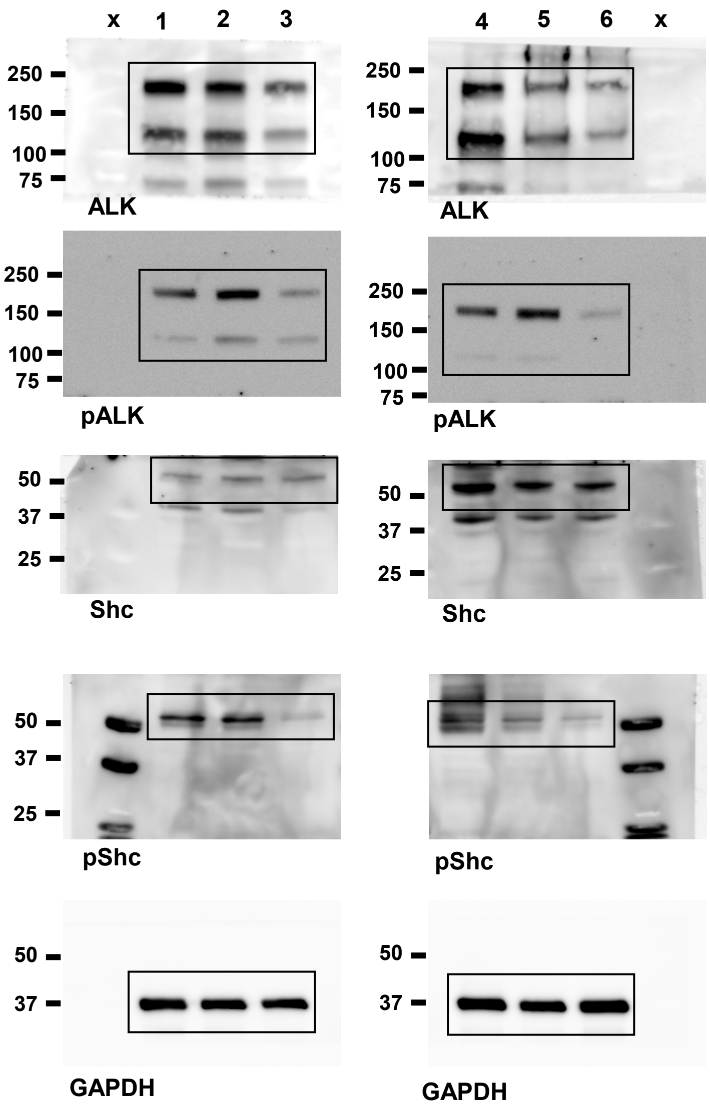

1. SH-SY5Y; DMSO
2. SH-SY5Y; Mismatch
3. SH-SY5Y; CCC-003
4. Kelly; DMSO
5. Kelly; Mismatch
6. Kelly; CCC-003

Fig 5C

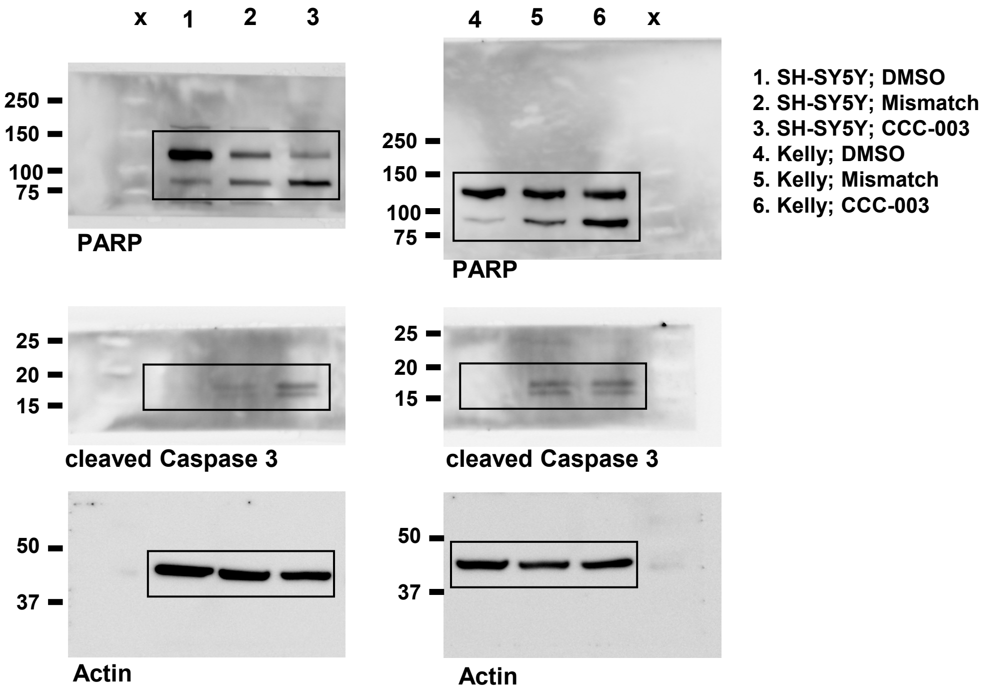
x
1
2
3
4
5
6
x
250
150
100
75
PARP
cleaved Caspase 3
25
20
15
Actin
50
37
1. SH-SY5Y; DMSO
2. SH-SY5Y; Mismatch
3. SH-SY5Y; CCC-003
4. Kelly; DMSO
5. Kelly; Mismatch
6. Kelly; CCC-003
